# A *Diaporthe* Fungal Endophyte From a Wild Grass Improves Growth and Salinity Tolerance of Tritordeum and Perennial Ryegrass

**DOI:** 10.3389/fpls.2022.896755

**Published:** 2022-05-27

**Authors:** Rufin M. K. Toghueo, Iñigo Zabalgogeazcoa, Eric C. Pereira, Beatriz R. Vazquez de Aldana

**Affiliations:** Plant-Microorganism Interaction Research Group, Institute of Natural Resources and Agrobiology of Salamanca (IRNASA-CSIC), Salamanca, Spain

**Keywords:** *Diaporthe*, plant growth promotion, salinity, fungi, endophyte, grasses, phytohormones, nutrient uptake

## Abstract

Some microbiome components can provide functions that extend the capabilities of plants, increasing the environmental adaptability and performance of holobionts. *Festuca rubra* subsp. *pruinosa* is a perennial grass adapted to rocky sea cliffs, where soil and nutrients are very limited, and exposure to salinity is continuous. This study aimed to investigate if a *Diaporthe* fungal endophyte belonging to the core microbiome of *Festuca rubra* roots could improve the performance of two agricultural grasses. In a greenhouse experiment, plants of tritordeum (*Triticum durum* x *Hordeum chilense*) and perennial ryegrass (*Lolium perenne*) were inoculated with *Diaporthe* strain EB4 and subjected to two salinity conditions (0 and 200 mM NaCl). Biomass production, mineral elements, proline, hormone profiles, antioxidant capacity, and total phenolic compounds were examined in plants, and fungal functions potentially related to the promotion of plant growth were determined. The inoculation with *Diaporthe* promoted plant growth of both grasses, increasing leaf biomass (84% in tritordeum and 29% in perennial ryegrass), root biomass, nutrient content (N, Ca, Mg, and Fe), and the production of indole 3-acetic acid, regardless of the salinity treatment. Improved growth and nutrient uptake might occur because *Diaporthe* produces several extracellular enzymes capable of recycling organic nutrient pools. In addition, the fungus produced indole 3-acetic acid *in vitro* and modulated the production of this phytohormone in the plant. Under salinity, the activity of *Diaporthe* ameliorated the stress, increasing proline, nutrient uptake in roots, gibberellins, and indole 3-acetic acid, which in turn results into improved growth. Thus, this fungus can transfer to alternative hosts some advantages useful at its original habitat.

## Introduction

The concept of biological individuality in plants is being replaced by their understanding as an assemblage of organisms or holobiont, composed by the plant itself plus its microbiome (Suarez, 2018). Microbiome components can provide functions that extend the capabilities of the plant, increasing the environmental adaptability and performance of holobionts (Vandenkoornhuyse et al., 2015). For example, improved nutrient acquisition, pest resistance, and thermal, drought or salinity tolerance in plants have been attributed to microbiome components (Rodriguez et al., 2009; Hardoim et al., 2015; Gupta et al., 2020; Liu et al., 2020).

Agrochemicals, plant breeding, and farm machines have been major technologies contributing to agricultural development during the 20th century, harnessing plant microbiomes might have an important role in the agriculture of the 21th century. Increased knowledge about microbiome functions related to plant performance is revealing attractive technological possibilities for agriculture (Ray et al., 2020; Pozo et al., 2021; Trivedi et al., 2021). For instance, studies of the microbiome of wild plants growing in inhospitable environments give an insight into microbial contributions to plant adaptation (Rodriguez et al., 2008; Furtado et al., 2019; Hosseyni Moghaddam et al., 2021; Pereira et al., 2021). A product of these studies could be the identification of microbes extending the performance of agricultural crops under stress factors present in the original environment of such microbes (Mahgoub et al., 2021). For instance, *Piriformospora indica*, one of the most widely studied root endophytes, promotes the growth a wide range of plants, partly due to enhanced root proliferation by indole 3-acetic acid production, which in turn results into better nutrient acquisition, and improves tolerance to a number of abiotic stresses (Gill et al., 2016). Soil salinity is recognized as one of the most critical constraints to crop productivity, preventing the cultivation of about 800 million hectares of land across the globe (Munns and Tester, 2008; FAO, 2021). Apart from natural salinity, soil salinization can be due to crop irrigation.

*Festuca rubra* subsp. *pruinosa* is a grass that grows in rocky maritime cliffs. In this windswept habitat, nutrient availability is very low because *Festuca* plants grow in rock fissures where soil is absent or very scarce, and salinity is high, due to seawater exposure (Castroviejo, 2021). The roots of these plants have a complex fungal endophytic microbiome, and a *Diaporthe* species is one of its most abundant components, occurring in more than 50% of the plants and all locations analyzed (Pereira et al., 2019). Other fungal endophytes of *Festuca* roots like *Periconia macrospinosa* and *Fusarium oxysporum* are known to contribute some beneficial functions to its host plant (Pereira et al., 2021). Thus, considering that *Diaporthe* is a component of the core endophytic microbiome of *Festuca rubra*, we expected that it could also have a function related to the habitat adaptation of its host plant. The genus *Diaporthe* and its *Phomopsis* anamorphs are constituted by pathogenic as well as by endophytic species (Udayanga et al., 2011; Gomes et al., 2013). Because of their economic impact, the best-known species are pathogenic, but reports about *Diaporthe* endophytes are increasing. Studies on the effects of endophytic *Diaporthe* species are scarce, but some reports show their involvement in plant growth promotion (Yang et al., 2014; Chithra et al., 2017; Vazquez de Aldana et al., 2021; da Silva Santos et al., 2022).

The objectives of this research were to evaluate the ability of a *Diaporthe* strain from *Festuca rubra* subsp. *pruinosa* to improve the growth and salinity tolerance of two agricultural grasses, and to determine fungal functions potentially related to plant growth promotion. Due to the abundance of a *Diaporthe* species in asymptomatic plants of *Festuca rubra* subsp. *pruinosa*, a grass growing in soils with low nutrient availability and saline conditions, our hypothesis was that *Diaporthe* could promote growth and improve salt tolerance when inoculated in other grass species. Two important agricultural crops were selected, tritordeum and perennial ryegrass (*Lolium perenne*). Tritordeum (× *Tritordeum* Ascherson et Graebner) is a hybrid grain cereal apt for human consumption, developed from a cross between durum wheat (*Triticum durum*) and *Hordeum chilense*, a wild barley species native to Chile and Argentina (Martín et al., 1999). In recent years, tritordeum has been introduced to European markets.^1^ Perennial ryegrass (*Lolium perenne*) is the most widely used species in Europe for sport fields and lawns, and it is also an important forage species.

## Materials and Methods

### Fungal Material Identification

*Diaporthe* strain EB4, originally isolated from roots of a *Festuca rubra* subsp. *pruinosa* plant (Pereira et al., 2019), was used in this study. This strain was selected in a previous screening where plants of *Lolium perenne* were inoculated with several *Diaporthe* strains and their growth evaluated in a greenhouse experiment (Supplementary Table S1).

The adscription of this strain within the *Diaporthe* genus was based on a multilocus phylogenetic analysis of the sequences of five genes (Gomes et al., 2013). The primers ITS4 and ITS5 were used to amplify the internal transcribed spacer (ITS) regions of the nuclear ribosomal genes (White et al., 1990). The primers EF1-728F/EF1-986R and CAL-228F/CAL-737R (Carbone and Kohn, 1999) were used to amplify part of the translation elongation factor 1α (TEF1) and the calmodulin (CAL) genes, respectively. The primers CYLH3F (Crous et al., 2004) and H3-1b (Glass and Donaldson, 1995) were used to amplify part of the histone H3 (HIS) gene, and the primers T1 (O’Donnell and Cigelnik, 1997) and Bt-2b (Glass and Donaldson, 1995) to amplify part of the β-tubulin gene (TUB). The amplification conditions were a start step of 2 min at 94°C, followed by 40 cycles of 30 s at 94°C, 1 min at 58°C for CAL, ITS and HIS, or 55°C for TEF-1 and TUB, and 1 min at 72°C, followed by a finishing step of 3 min at 72°C.

PCR amplicons were sequenced at the DNA sequencing service of the University of Salamanca (Spain). A concatenated sequence of the five loci (ITS, TUB, CAL, TEF1, HIS) was aligned with a set of 243 similar sequences belonging to 95 *Diaporthe* species obtained from Gomes et al. (2013). To determine the taxa closest to strain EB4, a maximum likelihood phylogenetic tree was made using MEGA v.7. After the clades close to strain EB4 were identified, new phylogenetic trees were made using the sequences of type strains of the species included in these clades. *Diaporthella corylina* was used as an outgroup, and the robustness of the tree topology was evaluated by 1,000 bootstrap replications.

### *In vitro* Characteristics of *Diaporthe* Strain EB4

#### Ammonium Production

Ammonium production was determined both qualitatively and quantitatively, as described by Cappuccino and Sherman (1998). For estimation, *Diaporthe* EB4 from a potato dextrose agar (PDA) culture was inoculated in test tubes containing 5 ml of peptone water and incubated for 7 days at 25°C under constant agitation (150 rpm). After incubation, 1 ml of the culture filtrate was transferred to a 1.5 ml microtube, and 50 μl of Nessler’s reagent (10% HgI_2_; 7% KI; 50% aqueous solution of 8 mM NaOH) were added. The development of a faint yellow color indicates a small amount of ammonium, and a deep yellow to brownish indicates a maximum production of ammonium. For the ammonium quantification, a multi-mode reader (Synergy^™^ 2 Multi-Mode Microplate Reader) measured the output at 450 nm using a standard curve of (NH_4_)_2_SO_4_ within the range of 78.1–625.0 μM. The assay was performed in duplicate.

#### Phosphate Solubilization

To evaluate the phosphate solubilizing ability of *Diaporthe* sp. EB4, a 6 mm plug of a PDA culture was plated onto freshly prepared Pikovskaya’s agar: 5.0 g Ca_3_(PO_4_)_2_; 0.5 g (NH4)_2_SO_4_; 0.1 g MgSO_4_·7H_2_O; 0.2 g NaCl; 0.2 g KCl; 3.0 mg FeSO_4_·7H_2_O; 3.0 mg MnSO_4_·H_2_O; 10.0 g glucose; 0.5 g yeast extract; 15.0 g agar in 1 l distilled water. Triplicate plates were incubated in the dark for 7 days at 25°C. After the incubation, the phosphate solubilization activity was characterized by the clear zone around the fungal colony (Shekhar Nautiyal, 1999).

#### Siderophore Production

The siderophore activity of *Diaporthe* EB4 strain was determined using the chrome azurol S (CAS) agar plate assay as described by Schwyn and Neilands (1987). Briefly, 6 mm diameter PDA plugs from the fungal isolate were plated on Blue Chrome Azurol A (CAS) agar medium freshly prepared. The plates were incubated in the dark at 25°C for 7 days. The color change in the medium from blue to yellow or orange indicates a positive result related to the siderophore production.

#### Indole Acetic Acid Production

The production of indole acetic acid (IAA) was determined quantitatively using both spectrophotometric and chromatographic (Szkop and Bielawski, 2013; Gang et al., 2019) assays. For the spectrophotometric quantification, the fungus was grown for 7 days at 25°C under constant agitation (150 rpm) in potato dextrose broth (PDB) supplemented with 1 mg/ml tryptophan. After the incubation period, the culture supernatant was mixed with Salkowski reagent in a 1:1 (v:v) ratio and incubated for 30 min in the dark, followed by reading the optical density at 530 nm using a multi-mode reader (Synergy^™^ 2 Multi-Mode Microplate Reader). The recorded absorbance was read off a standard curve prepared from pure IAA (Sigma-Aldrich, St. Louis, MO). The assay was performed in duplicate.

For HPLC quantification of IAA production (Szkop and Bielawski, 2013), a seven-day PDB culture of *Diaporthe* EB4 was extracted with ethyl acetate, and the extract obtained was dissolved in HPLC grade methanol (Scharlab, Spain), and centrifuged at 11,200 g for 5 min. The supernatant was filtered through a 0.45 μm nylon filter. The analysis of the extract was performed on a Waters 2,695 HPLC system, equipped with a fluorescence detector (Waters 2,475), using a C18 column (Waters Sunfire C18; 5 mm; 250 mm × 4.6 mm), at 40°C. The mobile phase consisted of methanol (60%) and 0.5% acetic acid (40%) in an isocratic flow of 0.7 ml/min. The excitation and emission wavelengths were 280 and 340 nm, respectively. A standard curve was prepared from IAA (Sigma-Aldrich, St. Louis, MO).

#### Enzymatic Activities

The ability of *Diaporthe* EB4 to produce amylase, cellulase, and protease was analyzed *in vitro* in the supplemented media described by Hankin and Anagnostakis (1975). The amylase activity was assessed on PDA containing 2% (w/v) soluble starch. After 7 days of incubation in the dark at 25°C, the plates were flooded for 15 min with a solution of 1% (w/v) iodine in 2% (w/v) potassium iodide. A clear zone surrounding the colony indicated amylase activity.

The fungal isolate was cultured on PDA supplemented with 0.5% carboxymethylcellulose (CMC) to determine the cellulase activity. After 7 days of incubation in the dark at 25°C, the plates were flooded with 0.2% (w/v) aqueous Congo Red and destained with 1 M NaCl for 15 min. The presence of a clear zone surrounding the colony indicated cellulase activity.

To determine the protease activity, the fungal isolate was grown on glucose yeast extract peptone agar medium supplemented with 0.4% gelatin for 7 days in the dark at 25°C. After the incubation, the degradation of the gelatin was noted by the formation of a clear zone around the fungal colony. The plates were then flooded with saturated aqueous (NH_4_)_2_SO_4_, which formed a precipitate that made the agar opaque and enhanced the clear zone around the colony.

### Inoculation of Plants and Experimental Design

To produce *Diaporthe* EB4 inoculum, 30 g of sugar beet pulp pellet mixed with 9.0 g CaCO_3_, 4.5 g CaSO_4,_ and 60 ml of water were autoclaved in wide-mouth glass bottles for 30 min at 121°C (Vazquez de Aldana et al., 2020). Each bottle of beet pulp substrate was inoculated with four plugs of mycelium from a potato dextrose agar (PDA) culture and incubated at room temperature (20°C–22°C) for 4 weeks.

To determine the effect of inoculation with *Diaporthe* EB4 on tritordeum and perennial ryegrass under salinity, a greenhouse experiment was designed for each plant species with two factors: inoculation with *Diaporthe* (inoculated or non-inoculated) and salinity treatment (0 or 200 mM NaCl). The use of 200 mM NaCl was based on preliminary assays about the response of tritordeum to salinity.

Seeds of tritordeum cv. Aucan (Cecosa Semillas, Spain) were sown in 200 ml pots filled with a substrate composed of peat and perlite (1,1 v/v) previously treated at 80°C for 24 h in a forced air oven. For the inoculation treatments, tritordeum seeds were sown in the same substrate containing fungal inoculum in a proportion inoculum:substrate 1:7 (v/v). Several seeds were sown in each pot and cleared to one seedling after emergence. Each treatment was replicated in 12 pots. Plants subjected to the salinity treatment were watered with a 50 and 100 mM NaCl solution on the first and third day, respectively, to avoid salt shock, and with a 200 mM NaCl solution from day 5 onwards during 3 weeks. After this time, the plants were harvested, roots carefully cleaned, and lyophilized. Dry biomass was recorded and samples were used for chemical analyses.

A similar experiment was performed with perennial ryegrass cv. Tivoli (DLF, Denmark) with the only difference that four plants per pot were grown. Roots of perennial ryegrass, being very thin and challenging to clean, were not collected for biomass analysis.

### Detection of Fungi in Inoculated Plants

The presence of *Diaporthe* in inoculated plants was diagnosed by direct isolation of the fungus from roots, and by microscopy. For this purpose, root samples of each inoculated tritordeum and perennial ryegrass plant were collected at harvest time. For isolation, a root sample was surface-disinfected and root pieces were placed in PDA plates following the method described by Pereira et al. (2019). For microscopy of fungal structures in roots, preparations of cleared (KOH) and non-cleared roots were stained with trypan blue, acid fuchsin, or aniline blue stain (Vierheilig et al., 2005).

### Measurements of Plant Physiological Parameters

#### Mineral Element Content

The concentration of mineral elements (Na, K, P, Ca, Mg, Fe, and Zn) was analyzed in six replicates of leaf samples of tritordeum and perennial ryegrass, and in roots of tritordeum. For that purpose, samples were calcined at 450°C for 8 h and ashes were dissolved in HCl:HNO_3_:H_2_O (1:1:8; Soto-Barajas et al., 2016). Na, K, P, Ca, Mg, Fe, and Zn contents were analyzed by inductively coupled plasma atomic emission spectroscopy (ICP-OES) in a Varian 720-ES (Agilent, United States) spectrometer. Total nitrogen content was analyzed in tritordeum plants by the Dumas combustion method in a CN analyzer (Leco CHN-628, United States).

#### Proline Content

Proline content was quantified in leaf samples (six replicates) using the spectrophotometric method described by Shabnam et al. (2016), adapted to 96-well plates in our laboratory. Approximately 15 mg of plant material were homogenized in 500 μl of 3% aqueous sulfosalicylic acid and kept for 10 min in ice. The mixture was centrifuged (10°C; 16,000 g; 10 min), and the supernatant was mixed with 250 μl of glacial acetic and 500 μl of ninhydrin, then the mixture was heated at 99°C for 40 min, and immediately cooled in ice. The mixture was centrifuged and an aliquot of 200 μl transferred to a 96-well plate where the absorbance was measured at 513 nm in a FLUOStar Omega plate reader (BMG Labtech, Germany). Standards of L(−) proline (Acrós Organics) were used for quantification.

#### Hormone Profile Analysis

The concentration of the cytokinins (CK) trans-Zeatin (tZ) and isopentenyladenine (iP), gibberellins (GA1 and GA3), indole-3-acetic acid (IAA), abscisic acid (ABA), salicylic acid (SA), and jasmonic acid (JA) were analyzed in tritordeum leaves (three replicates) according to Albacete et al. (2008) with some modifications. Briefly, 40 mg of plant material were homogenized in 1 ml of a cold (−20°C) extraction mixture of methanol/water (80/20, v/v). Solids were separated by centrifugation (20,000 *g*, 15 min) and re-extracted for 30 min at 4°C in 1 ml of the same extraction solution. Pooled supernatants were passed through a Sep-Pak Plus C_18_ cartridge (Waters, United States) to remove interfering lipids and plant pigments, and vacuum evaporated at 40°C to near dryness or until the organic solvent is removed. The residue was dissolved in 0.5 ml of a methanol/water (20/80, v/v) solution using an ultrasonic bath. The dissolved samples were filtered through 13 mm Millex nylon filters with 0.22 μm pore size (Millipore, Bedford, MA, United States).

Ten μl of filtered extract were injected in a UHPLC–MS system consisting of an Accela Series UHPLC coupled to an Exactive Mass Spectrometer (Thermo Fisher Scientific, Waltham, MA, United States) using a heated electrospray ionization (HESI) interface. Mass spectra were obtained using the Xcalibur software version 2.2 (Thermo Fisher Scientific, Waltham, MA, United States). For quantification of the plant hormones, calibration curves were constructed for each analyzed component (1, 10, 50, and 100 μg/l) and corrected for 10 μg/l deuterated internal standards. Recovery percentages ranged between 92% and 95%.

#### Ferric Reducing Antioxidant Potential Assay

The antioxidant capacity was determined in leaf samples from six replicates of each treatment using the ferric ion reducing antioxidant power (FRAP) method (Benzie and Strain, 1996). This method is based on the reduction of Fe^3+^2,4,6-tri(2-pyridyl)-*s*-triazine (TPTZ) complex (colorless) to Fe^2+^-tripyridyltriazine complex (blue colored), formed by the action of electron-donating antioxidants at low pH. The FRAP reagent was prepared by mixing 300 mM acetate buffer (pH = 3.6), a solution of 10 mM TPTZ in 40 mM HCl, and 20.35 mM FeCl_3_ at 10:1:1 (v/v/v). Five mg of each plant sample were extracted in 700 μl of 50% aqueous acetone for 30 min in an ultrasound bath at 8°C. The mixture was centrifuged and transferred to a 96-well plate wh 8 μl of sample, 8 μl of phosphate buffer saline, and 200 μl of FRAP reagent were added to each well. The absorbance was measured at λ = 593 nm after 30 min in a FLUOStar Omega (BMG Labtech, Germany). A standard curve was prepared using different concentrations of 6-hydroxy-2,5,7,8-tetramethylchroman-2-carboxylic acid (Trolox). All solutions used on the day of the experiment were freshly prepared. The results were expressed as μmol Trolox equivalent/g dry weight.

#### Total Phenolic Compounds Content

The content of total phenolic compounds in leaf samples was determined spectrophotometrically according to the Folin–Ciocalteu method, analyzing six replicates of each treatment (Ainsworth and Gillespie, 2007). A 100 μl aliquot of acetone extract of each sample, prepared as previously described for the FRAP assay, was mixed with 500 μl of Folin–Ciocalteu reagent (Scharlab Chemie SA). After 5 min, 400 μl of a 700 mM Na_2_CO_3_ solution was added, and after 60 min, absorbance was measured in a 96-well plate at 765 nm in a FLUOStar Omega (BMG Labtech, Germany). Gallic acid was used as a reference standard, and the results were expressed as μmol gallic acid equivalent/g dry weight.

### Statistical Analysis

The data sets were evaluated for the statistical assumptions of the ANOVA with the Shapiro–Wilk normality test and Brown–Forsythe equal variance test. Then, the effects of salinity and inoculation with *Diaporthe* on tritordeum and perennial ryegrass parameters were analyzed by two-way ANOVA. Differences between means were evaluated using Tukey’s test. Phytohormone data were analyzed with the Kruskal–Wallis test on ranks followed by Tukey’s test for multiple comparisons.

## Results

### *Diaporthe* Strain Identification

A preliminary phylogenetic analysis using 242 reference sequences from 95 *Diaporthe* species helped to identify the clades where strain EB4 was included. This allowed selecting a set of 13 *Diaporthe* species closely related to EB4, and a more restricted phylogenetic analysis was made using the sequences of type strains from these taxa (Supplementary Figure S1). In the maximum likelihood tree obtained, *Diaporthe* EB4 is most closely related to *Diaporthe scleotioides*, but according to the bootstrap values is not included within this taxon. Therefore, this strain appears to belong to a yet undescribed *Diaporthe* species.

### *In vitro* Activities of *Diaporthe* sp. EB4 Cultures

The *in vitro* evaluation of enzymatic and metabolic activities of *Diaporthe* EB4 cultures revealed some characteristics that could be associated with plant growth promotion (Table 1). *Diaporthe* EB4 produced ammonium in peptone water, and IAA in potato dextrose broth. On agar-supplemented media, *Diaporthe* produced siderophores and extracellular enzymes, such as amylase, cellulase, and protease, but was unable to solubilize phosphate.

**Table 1:** *In vitro* characteristics of *Diaporthe* strain EB4.

Parameter	Activity values
Siderophore production	2.85 ± 0.21 cm
Ammonium production	110.64 ± 8.77 μM
Indole acetic acid (IAA)	Spectrophotometric: 152.08 ± 3.24 μg/ml HPLC: 62.28 ± 1.54 μg/ml
Phosphate solubilization	Not detected
Cellulase	Positive
Amylase	Positive
Protease	Positive

### Detection of Fungi in Inoculated Plants

The isolation of *Diaporthe* from roots of inoculated plants was rare, occurring in less than one-fourth of the inoculated plants of each species, and in those cases only one or two root fragments produced *Diaporthe* isolates. No fungal structures were consistently observed by microscopy in roots of inoculated plants. Therefore, seems that the association of *Diaporthe* with tritordeum might be rhizospheric, and not endophytic.

### Effect of *Diaporthe* and Salinity on Plant Biomass Production

No visual symptoms of disease were observed in roots or leaves of plants inoculated with *Diaporthe* and/or subjected to salinity. This indicates that the strain EB4 is not pathogenic to tritordeum or perennial ryegrass.

**Figure 1:**
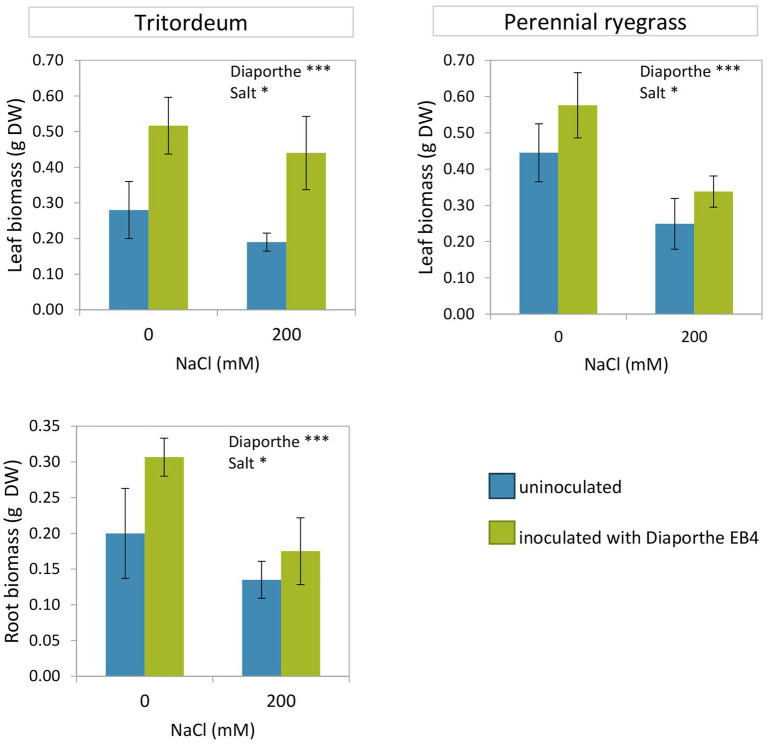
Leaf and root dry weight in tritordeum and perennial ryegrass plants not inoculated (**BLUE**) or inoculated with *Diaporthe* EB4 (**GREEN**), at two different salinity treatments (0 and 200 mM NaCl). Values are means ± SD (*n* = 12). Roots of perennial ryegrass were not collected and measured. Significant effects of “*Diaporthe* inoculation” and/or “salinity” are indicated as ^*^p < 0.05; ^***^p < 0.001.

Both inoculation with *Diaporthe* and salinity significantly affected the growth of tritordeum and perennial ryegrass, however, the interaction of both factors was not significant (Figure 1; Supplementary Tables S2, S3). The leaf biomass of both species increased in inoculated plants regardless of salinity. Compared to non-inoculated plants, *Diaporthe* increased leaf biomass of tritordeum by 84% at 0 mM NaCl, and by 131% at 200 mM NaCl (Figure 1). Similarly, *Diaporthe* inoculation increased leaf biomass of perennial ryegrass by 29% and 39% at 0 mM and 200 mM NaCl, respectively. The root biomass of tritordeum also increased in inoculated plants compared to non-inoculated at both salinity treatments. Compared to the 0 mM NaCl treatment, 200 mM NaCl significantly reduced the leaf biomass of tritordeum and perennial ryegrass by 20% and 42%, respectively, and the root biomass of tritordeum by 39% (Figure 1).

The comparison of both plant species showed that the relative increase in leaf growth due to *Diaporthe* was greater in tritordeum than in perennial ryegrass, and the decrease in growth due to salinity was greater in perennial ryegrass than in tritordeum.

### Effect of *Diaporthe* and Salinity on Plant Biochemical Parameters

#### Mineral Element Contents

**Figure 2:**
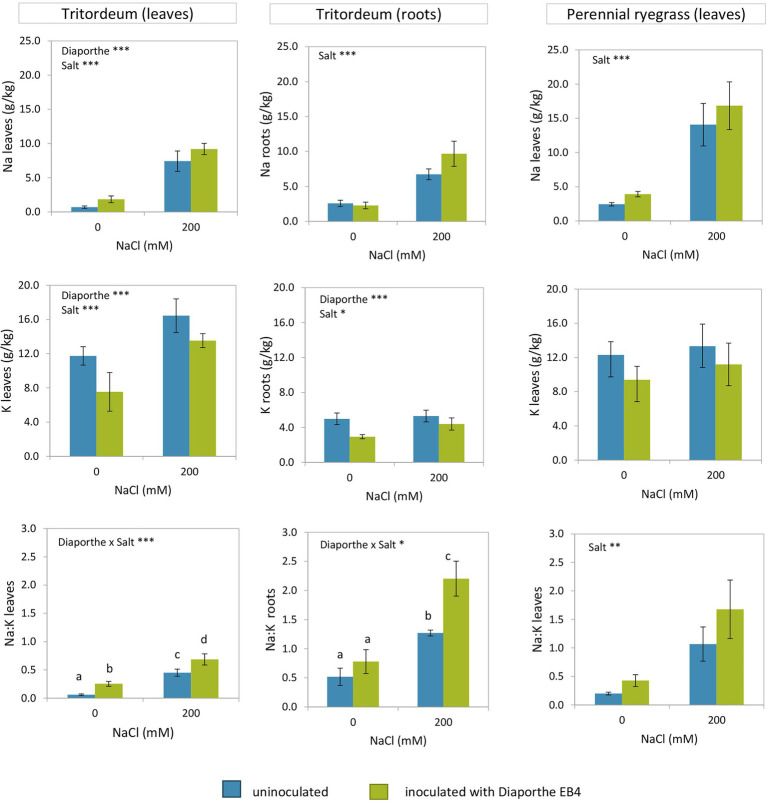
Sodium and potassium content in leaves and roots of tritordeum and leaves of perennial ryegrass plants not inoculated (**BLUE**) or inoculated with *Diaporthe* EB4 (**GREEN**), at two different salinity treatments (0 and 200 mM NaCl). Values are means ± SD (*n* = 6). When the interaction [*Diaporthe* inoculation × salinity] was significant, different means are indicated with different letters (Tukey test *p* < 0.05). Significant effects of “*Diaporthe* inoculation” and/or “salinity” are indicated as ^*^p < 0.05; ^**^p < 0.01; ^***^p < 0.001.

The Na^+^ content of tritordeum leaves was significantly affected by *Diaporthe* inoculation and salinity, but not by their interaction (Figure 2; Supplementary Table S2). In contrast, in perennial ryegrass only salinity had a significant effect on Na^+^ content (Figure 2; Supplementary Table S3). The increase in Na^+^ content due to salinity was greater in perennial ryegrass than in tritordeum. The ANOVA showed a significant effect of inoculation and salinity on the K^+^ content of tritordeum leaves (Supplementary Table S2). The K^+^ concentration increased in tritordeum plants exposed to 200 mM NaCl and decreased in plants inoculated with *Diaporthe* EB4 (Figure 2). In perennial ryegrass, the K+ content was not significantly affected by any factor.

In leaves of both plant species, the greatest Na^+^/K^+^ ratio was observed in inoculated plants exposed to salinity (Figure 2). In tritordeum a significant salinity × inoculation interaction indicated that *Diaporthe* affected this parameter mostly in the presence of salinity.

In roots of tritordeum Na^+^ increased at 200 mM NaCl regardless of *Diaporthe* (Figure 2) and the effect of inoculation was not significant. Root K^+^ followed the same trend as in leaves, that is, the concentration increased at 200 mM NaCl regardless of inoculation status and decreased in *Diaporthe*-treated plants respect to non-inoculated, regardless of salinity (Figure 2).

**Figure 3:**
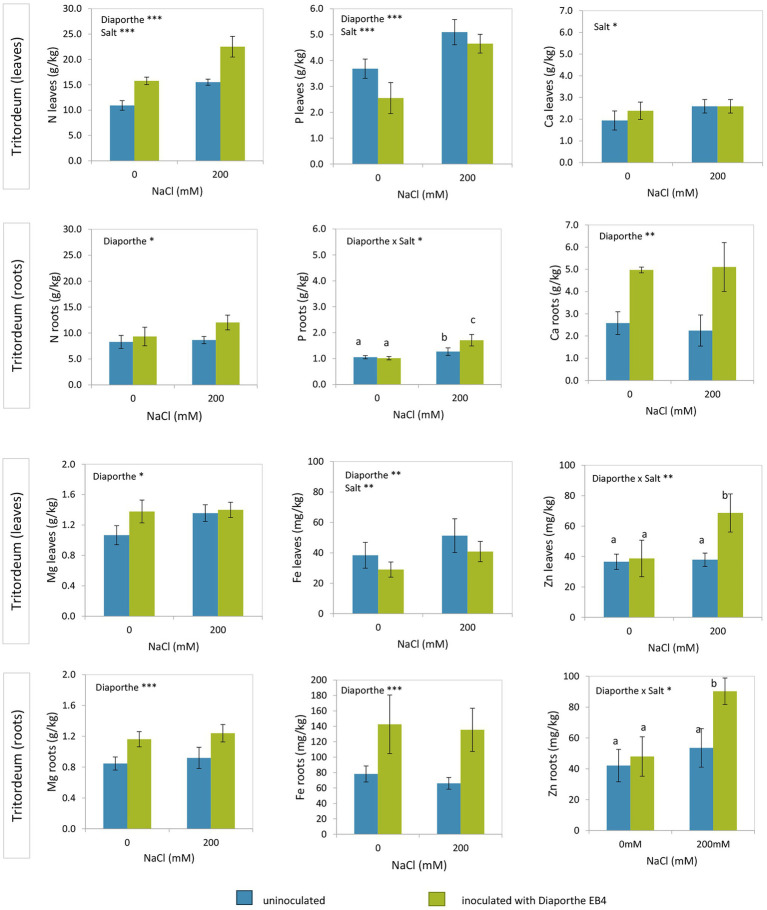
Total nitrogen, phosphorus, calcium, magnesium, iron, and zinc contents in leaves and roots of tritordeum plants not inoculated (**BLUE**) or inoculated with *Diaporthe* EB4 (**GREEN**), at two different salinity treatments (0 and 200 mM NaCl). Values are means ± SD (*n* = 6). When the interaction [*Diaporthe* inoculation × salinity] was significant, different means are indicated with different letters (Tukey test *p* < 0.05). Significant effects of “*Diaporthe* inoculation” and/or “salinity” are indicated as ^*^p < 0.05; ^**^p < 0.01; ^***^p < 0.001.

In tritordeum, leaf N increased in *Diaporthe*-inoculated plants regardless of salinity treatment and increased with salinity regardless of inoculation (Figure 3; Supplementary Table S2). P content was lower with *Diaporthe* than in non-inoculated plants regardless of salt treatment and increased at 200 mM compared to 0 mM NaCl regardless of inoculation. Ca in leaves increased at 200 mM compared to 0 mM NaCl regardless of inoculation, and the effect of *Diaporthe* inoculation was not significant. Mg content in leaves increased with *Diaporthe* inoculation respect to non-inoculated plants regardless of salinity, and the effect of salt treatment was not significant (Figure 3). Leaf Fe content decreased in inoculated plants compared to non-inoculated, regardless of salinity, and increased with salinity compared to 0 mM NaCl in both inoculation treatments. On the other hand, leaf Zn increased in *Diaporthe*-treated plants compared to non-inoculated at the 200 mM NaCl treatment, but the difference at 0 mM NaCl between inoculated and non-inoculated plants was not significant (Figure 3).

Nitrogen in roots of tritordeum increased with *Diaporthe* inoculation regardless of salinity (Figure 3; Supplementary Table S2). In roots of tritordeum the concentrations of Ca, Mg and Fe had a similar pattern of variation, increasing significantly in *Diaporthe*-inoculated plants compared to non-inoculated regardless of salinity, and concentrations were not affected by salt treatment (Figure 3). Regarding P and Zn in roots, *Diaporthe* increased their concentration at 200 mM NaCl, but differences at 0 mM NaCl between inoculation treatments were not significant.

#### Proline Content

**Figure 4:**
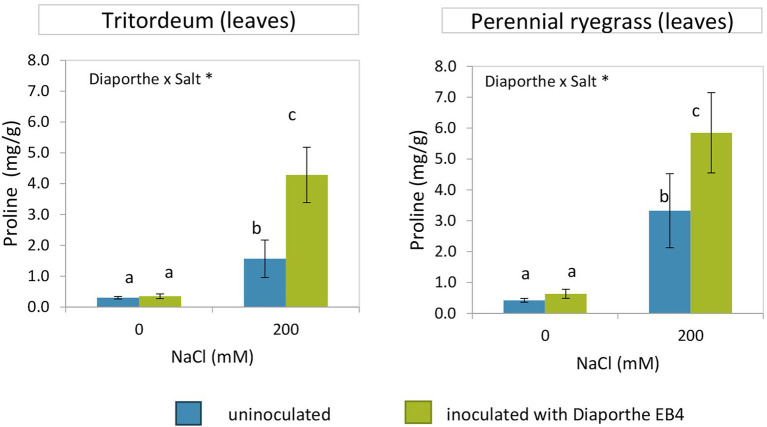
Proline content in leaves of tritordeum and perennial ryegrass plants not inoculated (**BLUE**) or inoculated with *Diaporthe* EB4 (**GREEN**), at two different salinity treatments (0 and 200 mM NaCl). Values are means ± SD (*n* = 6). When the interaction [*Diaporthe* inoculation × salinity] was significant, different means are indicated with different letters (Tukey test *p* < 0.05). Significant effects of “*Diaporthe* inoculation” and/or “salinity” are indicated as ^*^p < 0.05.

In both tritordeum and perennial ryegrass, a significant effect of inoculation, salinity, and their interaction was detected (Supplementary Tables S2, S3). In both plant species, the proline content increased significantly when plants were exposed to salinity, and such increase was much greater in inoculated plants (Figure 4). In the absence of salinity, *Diaporthe* did not change the proline content of any plant species. The mean proline content of leaves was greater in perennial ryegrass than in tritordeum.

#### Hormone Profiles

**Figure 5:**
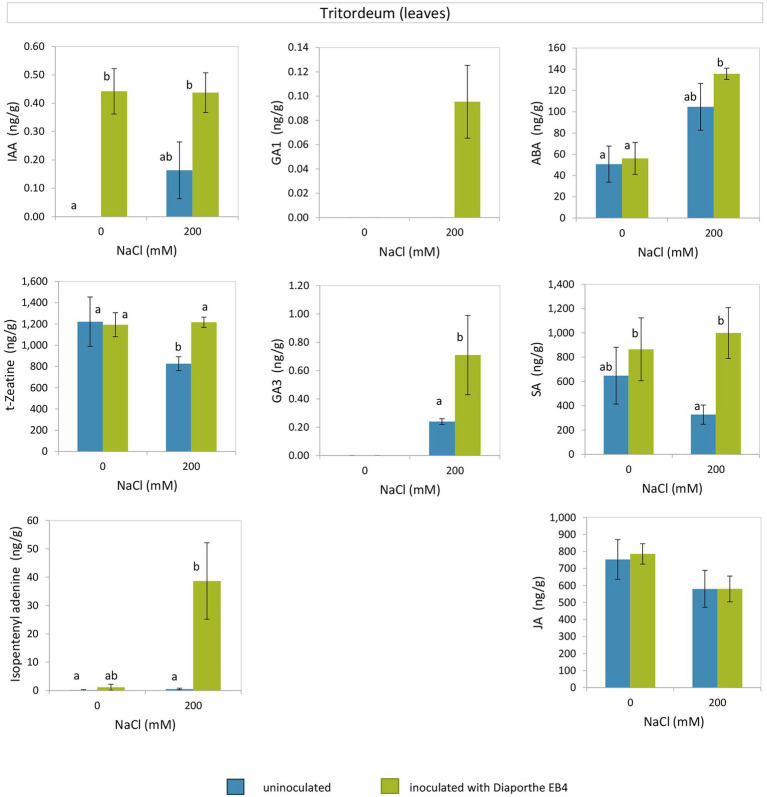
Phytohormones in leaves of tritordeum plants not inoculated (**BLUE**) or inoculated with *Diaporthe* EB4 (**GREEN**), at two different salinity treatments (0 and 200 mM NaCl): indole acetic acid (IAA), cytokinins (t-zeatin and isopentenyladenine), gibberellins (GA1 and GA3), abscisic acid (ABA), salicylic acid (SA), and jasmonic acid (JA). Values are means ± SD (*n* = 3). Different means are indicated with different letters (Tukey test *p* < 0.05).

*Diaporthe* inoculation caused a significant increase in IAA regardless of salinity, and this hormone was not detected in non-inoculated plants at 0 mM NaCl (Figure 5; Supplementary Table S4). The concentration of the cytokinin trans-Zeatin (t-Z) decreased at 200 mM NaCl in non-inoculated plants compared to the rest of treatments, and that of isopentenyladenine (iP) increased dramatically due to *Diaporthe* and salinity.

The gibberellin GA1 was detected only in inoculated plants at the salinity treatment, and GA3 was only detected in plants under salinity, with greater concentration in *Diaporthe*-inoculated plants than in non-inoculated (Figure 5). At 200 mM NaCl, the SA content was greater in *Diaporthe*-inoculated plants as compared to non-inoculated; however, at 0 mM NaCl differences between inoculation treatments were not significant. The ABA content did not vary between inoculation treatments at 0 and 200 mM NaCl; however, in *Diaporthe*-inoculated plants the ABA content was greater at 200 mM than at 0 mM NaCl. Regarding JA, differences among treatments were not significant.

#### Antioxidant Capacity and Total Phenolic Compounds Content

**Figure 6:**
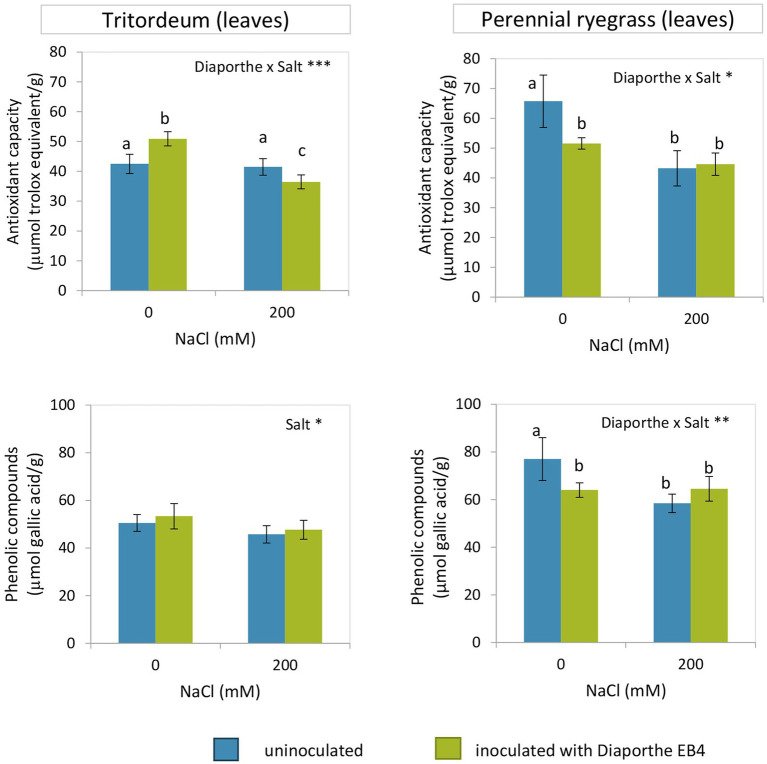
Antioxidant capacity and phenolic compounds content in leaves of tritordeum and perennial ryegrass plants not inoculated (**BLUE**) or inoculated with *Diaporthe* EB4 (**GREEN**), at two different salinity treatments (0 and 200 mM NaCl). Values are means ± SD (*n* = 6). When the interaction [*Diaporthe* inoculation × salinity] was significant, different means are indicated with different letters (Tukey test *p* < 0.05). Significant effects of “*Diaporthe* inoculation” and/or “salinity” are indicated as ^*^p < 0.05; ^**^p < 0.01; ^***^p < 0.001.

Inoculation with *Diaporthe* increased the antioxidant capacity of tritordeum plants at 0 mM NaCl, but decreased it under salinity (Figure 6; Supplementary Table S2). The phenolic compound content significantly decreased with salinity regardless of *Diaporthe* and was not affected by inoculation. However, in perennial ryegrass both the antioxidant capacity and phenolic compound content decreased in inoculated plants at 0 mM NaCl and did not change with inoculation at 200 mM NaCl (Figure 6; Supplementary Table S3).

## Discussion

In this work, we tested if a *Diaporthe* strain belonging to the core microbiome of a wild grass adapted to a nutrient-poor and saline habitat could be beneficial for two agricultural grasses. The results show that indeed, *Diaporthe* EB4 strain has the capability to promote the growth of tritordeum and perennial ryegrass in the presence as well as in the absence of salinity.

Based on a phylogenetic analysis, this strain seems to belong to a yet undescribed *Diaporthe* species. Nevertheless, further taxonomic studies including more *Diaporthe* strains isolated from with *Festuca rubra* subsp. *pruinosa* and their morphological characteristics will be necessary to describe a new *Diaporthe* species symbiotic with this grass.

### *Diaporthe*-Mediated Plant Growth Promotion

Our results showed that *Diaporthe* EB4 increased growth of tritordeum and perennial ryegrass in terms of leaf and root biomass, modifies the nutrient balance, and alter the level of some phytohormones, regardless of salinity treatment. Increased plant growth mediated by fungal symbiosis could occur because of improved plant nutrition, the production of phytohormones involved in growth regulation, or to a combination of both effects (Rai et al., 2014; Chanclud and Morel, 2016; Begum et al., 2019; Sarkar et al., 2021).

Some fungal root endophytes are reported to play an important role in the recycling and mineralization of organic nutrient pools in poor soils, like those found in Antarctica (Upson et al., 2009; Oses-Pedraza et al., 2020). Similarly, *Festuca rubra* subsp. *pruinosa* grows in a nutrient-poor rock substrate in sea cliffs. In such habitat, the nutrients found in dead roots and other plant debris could be recycled for further plant use by components of the root microbiome. Characteristics of *Diaporthe* EB4 cultures like the production of ammonium, siderophores, and extracellular enzymatic activities like cellulase, amylase, and protease, could support a nutrient cycling role similar to that of Antarctic endophytes (Hill et al., 2019; Oses-Pedraza et al., 2020). Ammonium is a preferential nitrogen source for plants, and *Diaporthe* EB4 cultures produced this compound in an assay where peptone, composed of peptides and amino acids, was used as a nitrogen source. In addition, a gelatin hydrolysis test confirmed the extracellular protease activity of this fungal strain. Therefore, *Diaporthe* EB4, like some Antarctic endophytes, can degrade protein to amino acids and ammonium, readily available plant nutrients, thus favoring plant growth (Upson et al., 2009; Hill et al., 2019). The increase in total nitrogen detected in leaves and roots of inoculated plants would support this hypothesis. Other hydrolytic enzymes produced by *Diaporthe*, like cellulase and amylase may also improve nutrient availability *via* the degradation of the plant cell walls from dead plant tissues and other organic constituents of the soil (van den Brink and de Vries, 2011). The enzymatic degradation of cellulose and other cell wall polysaccharides can produce glucose and other monosaccharides, which can be absorbed by plant roots, as well as by rhizospheric microorganisms (Yamada et al., 2011). The saprobic capability of *Diaporthe* EB4 further supports its possible function in nutrient cycling. This strain grew profusely in the beet pulp medium used to prepare its inoculum, and another *Diaporthe* strain showed the greatest saprobic capability among several root endophytes grown in this polysaccharide-rich medium (Vazquez de Aldana et al., 2020). Therefore, degradation of organic compounds as a result of the saprobic growth of *Diaporthe* on plant remains could improve the availability of plant nutrients in the rhizosphere.

Another beneficial effect for plants could be derived from the production of fungal siderophores, chelator agents with specificity for binding insoluble Fe^3+^ (Ahmed and Holmström, 2014). *Diaporthe* EB4 cultures produced siderophores, and this might be related to the significantly greater accumulation of Fe observed in roots of inoculated plants, regardless of salinity treatment. Fe is essential for chlorophyll biosynthesis and photosynthesis (Ahmed and Holmström, 2014). The greater Fe accumulation in roots than in leaves could occur because plants acquire Fe from the rhizosphere through the root system, but the translocation to leaves is regulated by the response to a Fe deficiency signal, as reported in barley (Tsukamoto et al., 2009). Other essential nutrients like Mg, Ca, and Zn also increased in response to *Diaporthe* inoculation.

There is no strong evidence for *Diaporthe* EB4 being an endophyte in tritordeum or ryegrass. Neither the fungus was consistently isolated, nor fungal structures were observed in roots. Therefore, it is possible that the beneficial activity observed in inoculated plants is derived from an epiphytic association, where the fungus is limited to the rhizospheric space. In contrast, *Diaporthe* strains were frequently isolated from roots of *Festuca rubra* subsp. *pruinosa*, the original host of the EB4 strain (Pereira et al., 2019). Many *Diaporthe* species are host generalists (Gomes et al., 2013), and the capability of EB4 to infect root tissues might be greater in its original *Festuca* host than in our agricultural grasses. Nevertheless, in a way similar to what we have observed here, some dark septate endophytes enhance plant growth without clear evidence of root colonization (Newsham, 2011; Yakti et al., 2018).

Coupled with increased nutrient availability, the production of IAA by *Diaporthe* itself, as well as the increased content of this hormone observed in inoculated plants, could explain the increased biomass observed. Similarly, it has been suggested that the growth promotion in rice stimulated by *Phomopsis* (=*Diaporthe*) may be due to its high IAA production capacity (Chithra et al., 2017). A recent investigation reveal that the inoculation of common bean plants with endophytic fungi surpassed the effect of the exogenously applied hormones (IAA), which resulted in the improvement of plant biomass, photosynthetic pigments, carbohydrate and protein contents (Ismail et al., 2021). IAA is involved in vascular development and cell elongation, enhancing plant growth and inducing lateral root formation (Chanclud and Morel, 2016; Egamberdieva et al., 2017). The stimulation of lateral roots improves nutrient uptake, and consequently increases plant growth. Auxins are also involved in plant–fungal symbiotic interactions, for instance, they are required for the colonization of mycorrhizal fungi (Chanclud and Morel, 2016).

### Plant Tolerance to Salinity Mediated by *Diaporthe*

Soil salinity inhibits plant growth for two reasons: first, a high concentration of salt reduces the water uptake capability of plants (osmotic stress), and second, an excessive salt concentration within the plant can be toxic (ionic stress; Munns and Tester, 2008). Plants have a rapid response to an increase in external osmotic pressure, and a slower response to the internal Na^+^ accumulation. In our experiments, salinity reduced tritordeum and perennial ryegrass growth. However, *Diaporthe* helped plants to overcome the stress, improving biomass accumulation and altering related physiological parameters, such as proline and gibberellins.

As expected, proline increased in both plant species in response to salinity, but the concentration of this osmolyte further increased due to *Diaporthe*. Therefore, although inoculated plants showed a greater Na^+^ leaf uptake than their non-inoculated counterparts, increased proline synthesis might compensate the deleterious effect of this ion. Due to the Na^+^ sequestration in cell vacuoles, an osmotic adjustment of the cytoplasm is needed to maintain cell turgor, and this is supposedly achieved by the active accumulation of organic solutes (e.g., proline and sugars) and inorganic ions (mainly K^+^ and Cl^−^; Munns and Tester, 2008; Zhao et al., 2020). In addition to osmotic adjustment, proline has multiple functions stabilizing the structure of proteins and scavenging ROS (Acosta-Motos et al., 2017). In accordance with our results, other endophytes have been shown to enhance proline synthesis in plants, improving salt stress tolerance (Zhang et al., 2016; Bouzouina et al., 2021; Mahgoub et al., 2021). In contrast, in *Festuca rubra* subsp. *pruinosa*, the original host of *Diaporthe* EB4, proline synthesis in response to salinity was independent of the symbiosis with three different fungal endophytes, *Epichloë festucae*, *Periconia macrospinosa*, and *Fusarium oxysporum* (Pereira et al., 2021). The fact that this is a halophytic grass might be related to this.

A high Na^+^ content in the soil can induce an iron deficiency or imbalance due to competition for the uptake of nutrients, such as K^+^ and Ca^2+^, thereby affecting plant physiological parameters (Hu and Schmidhalter, 2005; Evelin et al., 2019). Na^+^ accumulation in the plant due to salinity did not decrease the K^+^ content, which increased in tritordeum and did not change significantly in perennial ryegrass. However, *Diaporthe* had a negative effect on K^+^ content in both plant species, and thus the Na^+^/K^+^ ratio increased in inoculated plants. Fungi, such as *Aspergillus aculeatus*, *Piriformospora indica*, or *Fusarium*, are able to increase K^+^ in plants under salinity improving the Na^+^/K^+^ homoeostasis maintaining a low ratio (Xie et al., 2017; Ghorbani et al., 2019). Other *Festuca rubra* endophytes also have a positive effect on the adaptation to salinity of their host plants. For instance, *Periconia macrospinosa* has similar effects in its host plant to the ones here observed with *Diaporthe* in tritordeum and ryegrass under salinity: *Festuca rubra* inoculated with *Periconia* showed increased biomass and Na^+^ content, and decreased K^+^ content respect to uninoculated plants (Pereira et al., 2021). *Fusarium oxysporum*, the most abundant component of the *Festuca* root microbiome, promoted the growth of its host plant under salinity, but in contrast to *Periconia* and *Diaporthe*, the Na^+^ content was significantly reduced in inoculated plants, and K^+^ remained unchanged. This suggests that Na^+^, K^+^, and their ratio, can be altered in plants experiencing symbiotic growth promotion.

Ca^2+^ availability could be seriously reduced under salinity because Na^+^ readily displaces Ca^2+^ from its extracellular binding sites. This could be detrimental for plant growth because Ca^2+^ plays an essential role in the structure and function of plant membranes and in proline metabolism (Hu and Schmidhalter, 2005; Iqbal et al., 2014). However, we found that under salinity Ca^2+^ increased slightly in tritordeum leaves, but its accumulation in roots greatly increased in inoculated plants (under saline and non-saline conditions). Thus, a *Diaporthe* mediated improvement in Ca^2+^ uptake that accumulates in roots, might improve the ionic homeostasis, and be related to cellular stability and the increased proline accumulation and salinity tolerance observed. Exogenous application of CaCl_2_ has been found to increase proline content, thus mitigating salt stress (Khan et al., 2010). In addition to Ca^2+^, *Diaporthe* also improved Mg^2+^ in roots (regardless of salinity) and Zn in roots of plants under salinity. These nutrients are important in plants, regulating various metabolic pathways involved in abiotic stress tolerance (Khan et al., 2018). The fact that those nutrients accumulate in roots and are not mobilized to shoots could be due to an adequate concentration in shoots.

Phytohormones play an important role in plant tolerance to stress by modulating the physiological responses and defense systems of the plant. ABA synthesis is one of the fastest responses to abiotic stress factors, causing stomatal closure, thereby reducing water loss *via* transpiration (Chaves et al., 2009; Chanclud and Morel, 2016). As expected, ABA increased in tritordeum exposed to salinity, and *Diaporthe* enhanced this response. Similarly, GAs increased due to *Diaporthe* under salinity, and this can help plant growth maintenance during stress conditions. In fact, exogenous application of GA to plants mitigated saline stress, and the same effect was observed when fungal endophytes producing GA were inoculated (Tuna et al., 2008; Khan et al., 2012; Hamayun et al., 2017). Among other effects, GAs can influence ion uptake and proline metabolism (Iqbal et al., 2014; Al-Harthi et al., 2021). We did not analyze if *Diaporthe* EB4 can produce GAs *in vitro*, but this occurs in fungi, such as *Aspergillus, Fusarium, Porostereum spadiceum*, or *Paecilomyces formosus* (Khan et al., 2012; Waqas et al., 2012; Hamayun et al., 2017; Bilal et al., 2018). SA is involved in defense signaling against biotrophic pathogens (Pieterse et al., 2014), but also improves plant stress tolerance through modulation of antioxidative enzymes and increasing the proline metabolism (Misra and Saxena, 2009; Khan et al., 2015). We found that in response to salinity SA decreased in non-inoculated plants but it was maintained at non-stress levels in *Diaporthe*-inoculated plants. Although SA is involved in the modulation of antioxidative enzymes, the increase in SA mediated by *Diaporthe* under salinity was not reflected in the total antioxidant capacity of tritordeum plants, which decreased under salinity. However, this could be related to the increase in proline, which also has a role in scavenging reactive oxygen species (Iqbal et al., 2014). Association with fungal endophytes has been reported to enhance the effectiveness of the plant host antioxidative system by manipulating the production or activity of antioxidants under stress (White and Torres, 2010).

In conclusion, our study shows that a *Diaporthe* strain isolated from roots of an halophytic grass promotes the growth of two agricultural grasses, tritordeum and perennial ryegrass, by increasing nutrient uptake and the production of indole 3-acetic acid. Improved growth and nutrient uptake might be due to the fact that *Diaporthe* produces several extracellular enzymes capable of degrading organic nutrient pools, and siderophores that improve iron uptake. In connection with improved nutrient availability, *Diaporthe* increased the IAA content in the plant, which is related to root growth promotion. Under salinity, *Diaporthe* EB4 ameliorated stress through accumulation of GA and IAA in plants, increased proline synthesis, and nutrient uptake in roots, which subsequently led to improved tritordeum and perennial ryegrass growth. These findings might provide a foundation for further research about this possibly new *Diaporthe* species from a halophytic grass as a natural plant growth-promoting agent in agricultural grasses.

## Data Availability Statement

The original contributions presented in the study are included in the article/Supplementary Material, further inquiries can be directed to the corresponding author.

## Author Contributions

RT and EP performed experiments and analyses. All authors designed the experiments, worked in the analysis of data and writing of the manuscript, and contributed to the article and approved the submitted version.

## Funding

This research was supported by grant AGL2016-76035-C21R funded by MCIN/AEI/10.13039/501100011033 and “ERDF A way of making Europe,” grant PID2019-109133RB-I00 funded by MCIN/AEI/10.13039/501100011033; by the European Union’s H2020 research and innovation program under the Marie Sklodowska-Curie grant agreement no. 676480; and by project “CLU2019-05—IRNASA/CSIC Unit of Excellence” funded by the Junta de Castilla y León and co-financed by EU (ERDF “Europe drives our growth”). RT was supported by grant FJC2018-03857-I funded by MCIN/AEI/10.13039/501100011033 and by “ESF Investing in your Future.”

## Conflict of Interest

The authors declare that the research was conducted in the absence of any commercial or financial relationships that could be construed as a potential conflict of interest.

## Publisher’s Note

All claims expressed in this article are solely those of the authors and do not necessarily represent those of their affiliated organizations, or those of the publisher, the editors and the reviewers. Any product that may be evaluated in this article, or claim that may be made by its manufacturer, is not guaranteed or endorsed by the publisher.

## Footnotes

^1^ www.tritordeum.com
