# Supplementary material for: A Diaporthe Fungal Endophyte From a Wild Grass Improves Growth and Salinity Tolerance of Tritordeum and Perennial Ryegrass
Source: Front Plant Sci. 2022 May 27;13:896755. doi: 10.3389/fpls.2022.896755 (PMC9198640; doi:10.3389/fpls.2022.896755)
Supplement: Supplementary file 1 [file Data_Sheet_1.PDF]

Supplementary Material

Supplementary Table S1

Table S1. Results from a preliminary experiment used to select *Diaporthe* EB4 strain. Leaf biomass variation (%) of perennial ryegrass inoculated with different strains of *Diaporthe* isolated from *Festuca rubra* subsp. *pruinosa* with respect to uninoculated plants, at two different salinity treatments (0 and 200 mM NaCl). Values are obtained from dry matter means of uninoculated and inoculated plants (n=12).

Strain	Change in leaf biomass respect to uninoculated control plants (%)	
	0 mM NaCl	200 mM NaCl
Diaporthe EB4	82.67	101.06
Diaporthe S32	67.05	25.53
Diaporthe CP36	48.01	6.03
Diaporthe S69	45.57	95.66
Diaporthe S129	-60.87	-46.61

Supplementary Table S2

Table S2. Results of normality test (Shapiro-Wilk) equal variance test (Brown-Forsythe), and two-way analysis of variance results showing the effect of inoculation with *Diaporthe* EB4, salinity and their interaction on tritordeum parameters. Numbers in red mean that the factor significantly affects the variable.

	Normality test	Equal variance test	*Diaporthe* inoculation		Salinity		*Diaporthe* × Salinity	
	P	P	F	P	F	P	F	P
Leaf dry weight	0.321	0.239	59.34	<0.001	6.960	0.016	0.045	0.835
Root dry weight	0.646	0.123	17.05	0.001	30.67	<0.001	3.524	0.075
Na leaves	0.155	0.076	14.88	0.001	342.7	<0.001	0.689	0.417
K leaves	0.528	0.565	25.95	<0.001	58.76	<0.001	0.833	0.373
Na:K leaves	0.102	0.187	63.08	<0.001	232.8	<0.001	232.8	<0.001
N leaves	0.215	0.657	87.15	<0.001	79.70	<0.001	2.918	0.107
P leaves	0.184	0.453	12.10	<0.001	59.87	<0.001	2.335	0.143
Ca leaves	0.194	0.446	1.342	0.261	4.995	0.038	1.362	0.258
Mg leaves	0.139	0.648	4.238	0.050	3.268	0.086	2.424	0.136
Fe leaves	0.445	0.674	8.576	0.009	13.33	0.002	0.025	0.876
Zn leaves	0.766	0.128	13.90	0.001	12.51	0.002	10.47	0.004
Na root	0.080	0.649	3.112	0.105	59.36	<0.001	4.675	0.054
K root	0.999	0.566	23.01	<0.001	8.381	0.015	3.393	0.093
Na:K root	0.324	0.072	15.75	0.002	51.34	<0.001	5.198	0.044
N root	0.832	0.724	6.262	0.037	3.160	0.119	1.876	0.213
P root	0.285	0.554	6.322	0.029	32.21	<0.001	8.966	0.012
Ca root	0.209	0.161	16.52	0.002	0.023	0.880	0.132	0.723
Mg root	0.145	1.000	29.54	<0.001	1.663	0.224	0.001	0.972
Fe root	0.519	0.081	14.09	0.003	0.293	0.599	0.019	0.892
Zn root	0.365	0.864	13.02	0.004	20.73	<0.001	6.834	0.024
Proline	0.123	0.075	7.995	0.011	28.21	<0.001	7.447	0.013
Antioxidant capacity	0.496	0.270	1.861	0.193	38.22	<0.001	28.85	<0.001
Total phenolics	0.671	0.907	1.297	0.273	6.319	0.024	0.043	0.839

Supplementary Table S3

Table S3. Results of normality test (Shapiro-Wilk), equal variance test (Brown-Forsythe) and two-way analysis of variance results showing the effect of inoculation with *Diaporthe* EB4, salinity and their interaction on perennial ryegrass parameters. Numbers in red mean that the factor significantly affects the variable.

	Normality test	Equal variance test	*Diaporthe* inoculation		Salinity		*Diaporthe* × Salinity	
	P	P	F	P	F	P	F	P
Leaf dry weight	0.650	0.357	14.85	<0.001	69.91	<0.001	1.110	0.297
Na leaves	0.141	0.076	1.025	0.341	34.06	<0.001	0.009	0.766
K leaves	0.663	0.684	3.229	0.110	1.012	0.344	0.073	0.794
Na:K leaves	0.069	0.430	2.371	0.162	15.14	0.005	0.492	0.503
Proline	0.650	0.357	10.19	0.013	65.41	<0.001	7.891	0.023
Antioxidant capacity	0.296	0.245	5.014	0.045	26.78	<0.001	7.445	0.018
Total phenolics	0.369	0.442	1.449	0.252	9.902	0.008	11.12	0.006

Supplementary Table S4

Table S4. Results of normality test (Shapiro-Wilk), equal variance test (Brown-Forsythe) and Kruskal-Wallis one way analysis of variance on ranks showing the effect of the [*Diaporthe* inoculation × Salinity] treatments: uninoculated-0mM NaCl; *Diaporthe*-0mM NaCl; uninoculated-200mM NaCl; *Diaporthe*-200mMNaCl, on tritordeum phytohormones. Numbers in red mean that the factor significantly affects the variable.

	Normality test	Equal variance test	Kruskal-Wallis
	P	P	P
trans-Zeatine (CK)	0.447	0.333	0.048
Isopentenyl adenine (CK)	<0.050	0.067	0.039
Giberellins GA1	<0.050	1.000	N/A*
Giberellins GA3	<0.050	0.271	0.050
Indol acetic acid (IAA)	0.663	0.114	0.038
Abscisic acid (ABA)	0.996	0.634	0.018
Salicylic acid (SA)	0.746	0.261	0.045
Jasmonic acid (JA)	0.120	0.679	0.077

(*) Not applicable: GA1 was only detected in the *Diaporthe*-200mMNaCl treatment

Supplementary Figure S1

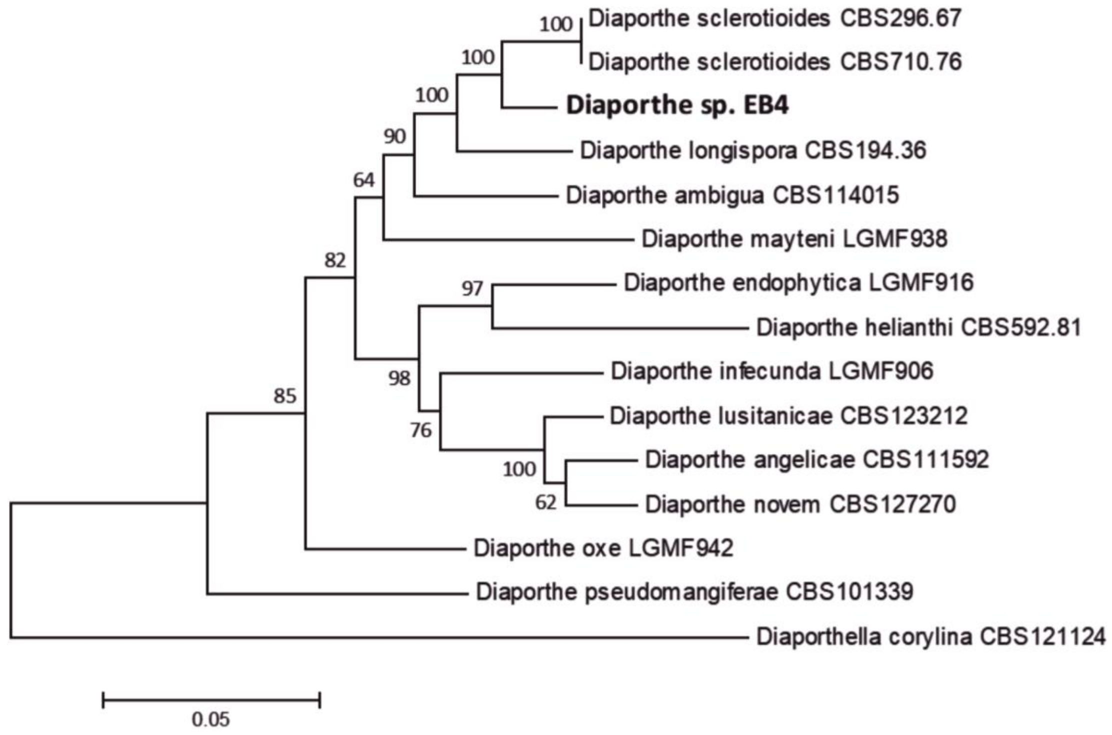

Figure S1. Maximum likelihood phylogenetic analysis of the combined 5-gene sequence alignment (ITS, TUB, CAL, TEF-1, and HIS) of *Diaporthe* strain EB4 and closely related *Diaporthe* species. Bootstrap values are shown at the nodes and the scale bar represents the expected changes per site. The tree was rooted to *Diaporthella corylina*.
